# Supplementary material for: The molecular basis of chaperone-mediated interleukin 23 assembly control
Source: Nat Commun. 2019 Sep 11;10:4121. doi: 10.1038/s41467-019-12006-x (PMC6739322; doi:10.1038/s41467-019-12006-x)
Supplement: Supplementary file 1 — Supplementary Information [file 41467_2019_12006_MOESM1_ESM.pdf]

Supplementary Information

The molecular basis of chaperone-mediated interleukin 23 assembly control

S. Meier et al.

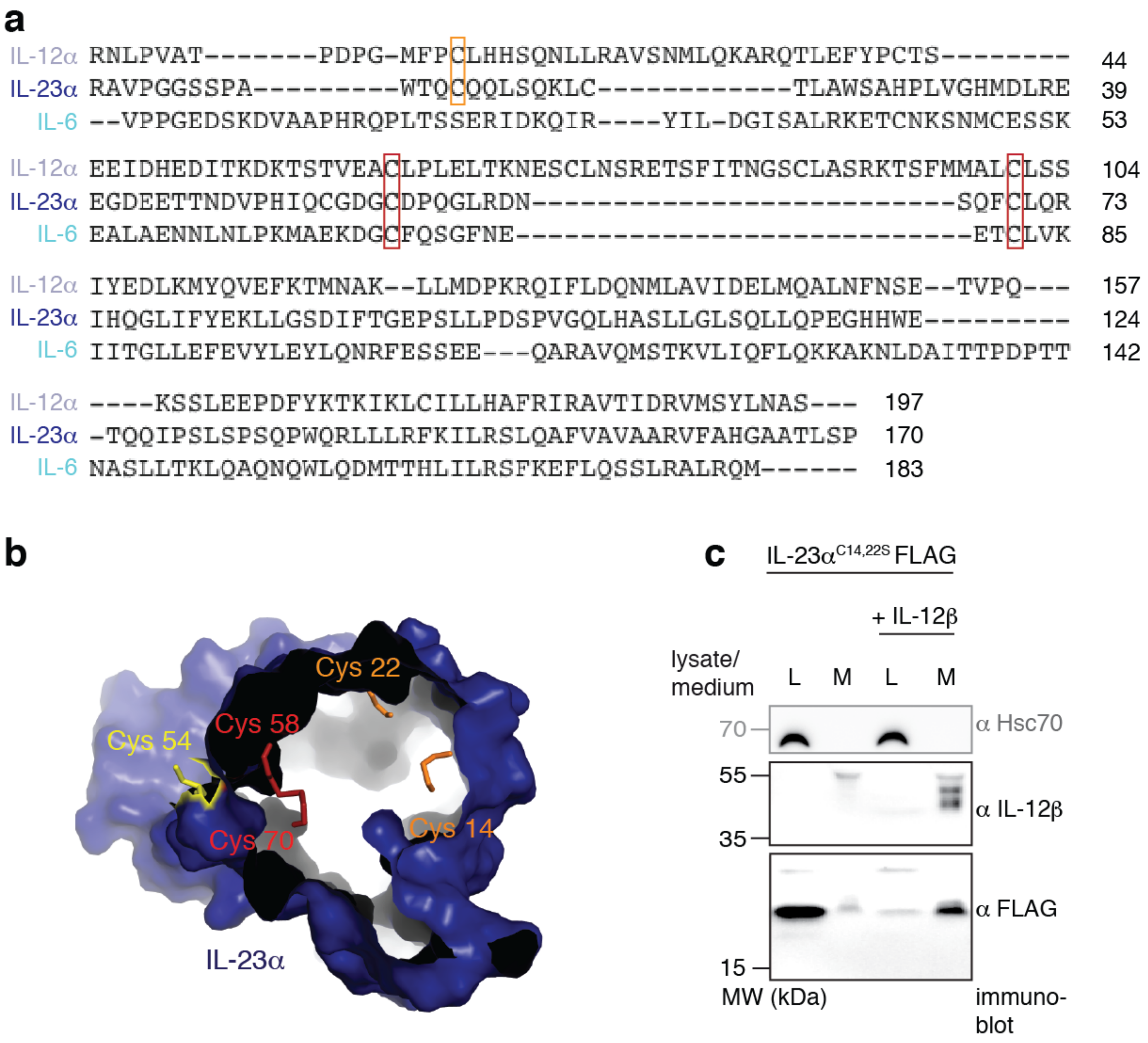

Supplementary Fig. 1 Cysteine conservation between IL-12α, IL-23α and IL-6. **a** Sequence alignment of human IL-12α, IL-23α and IL-6. Cysteines forming a conserved disulfide bond (see Fig. 1**e**) are highlighted in red. IL-23α C14 and its corresponding cysteine in IL-12α are framed in orange. **b** Surface model of IL-23α (with the same color code as in Fig. 1**d**) shows solvent-accessibility of indicated cysteines. **c** Secretion behavior of FLAG-tagged IL-23α^{C14,22S} in the presence or absence of IL-12β. Hsc70 served as loading control.

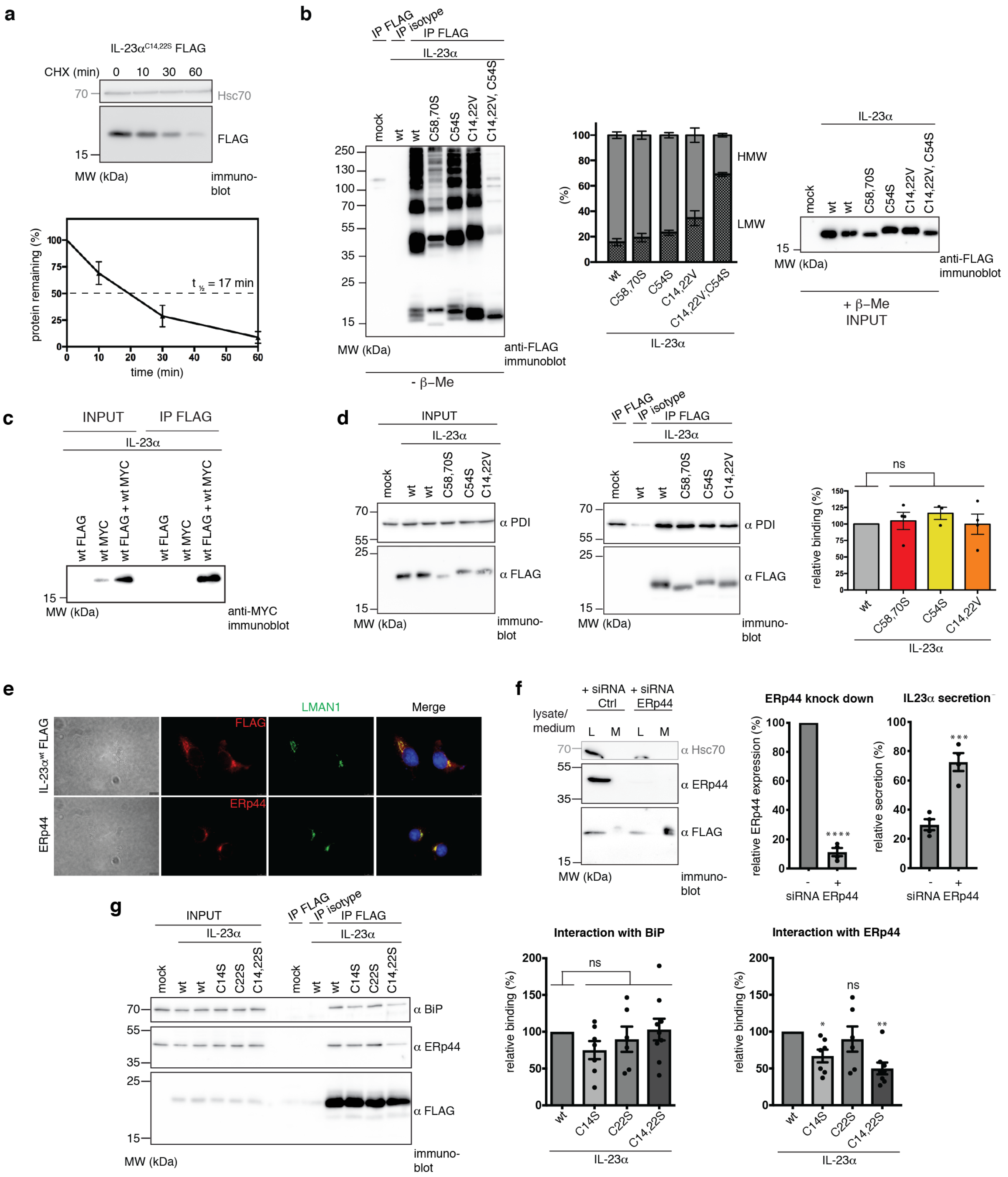

Supplementary Fig. 2 ER chaperone interaction of IL-23α. **a** Time course of degradation of IL-23α^{C14,22S} upon inhibition of translation with cycloheximide (CHX) for up to 60 min. Hsc70 served as loading control. Bottom: Quantification of protein turnover is shown; data are the mean ±SEM of at least four independent experiments. Source data are provided as a Source Data File. **b** Redox status of FLAG-tagged IL-23α cysteine mutants. Samples were treated with NEM to conserve the redox species,

immunoprecipitated and analyzed by non-reducing SDS-PAGE and anti-FLAG immunoblots. A quantitative analysis (middle) indicates the percentage of high molecular weight (HMW) and low molecular weight (LMW) IL-23α species (N=3±SEM). LMW species (black) were defined as smaller than 25 kDa, HMW species (grey) as larger than 25 kDa. Source data are provided as a Source Data File. Right: Input samples analyzed under reducing conditions. **c** Dimerization of FLAG- and MYC-tagged IL-23α. Samples were treated with NEM, immunoprecipitated against FLAG-tag where indicated and analyzed by reducing SDS-PAGE and anti-MYC immunoblots. **d** Immunoblot analysis of co-immunoprecipitated endogenous protein disulfide isomerase (PDI) with FLAG-tagged IL-23α^{wt}, IL-23$\alpha^{C58,70S}$, IL-23α^{C54S} and IL-23$\alpha^{C14,22V}$. Relative intensities of each band (±SEM) were calculated for three to four independent experiments and normalized to the IL-23α^{wt} signal which was set to 100% relative binding. Statistical significance was calculated using a two-tailed unpaired t-test. ns = not significant. **e** Immunofluorescence analysis of endogenous ERp44 and FLAG-tagged IL-23α^{wt}. Either COS-7 cells transfected with IL-23α^{wt} (top) or untransfected COS-7 cells (bottom) were immunostained for FLAG-tagged IL-23α^{wt} (red) or endogenous ERp44 (red), respectively. Additionally, cells were immunostained for LMAN1 (green) as an ER-Golgi intermediate compartment (ERGIC) marker, and nuclei were stained with DAPI (blue). ERp44 immunofluorescence and FLAG immunofluorescence signals from IL-23α^{wt} as well as LMAN1 and nuclei are displayed as central cell planes. Depicted images are representative of cells from at least three different biological replicates. Scale bars (left DIC images) correspond to 10 μm. **f** siRNA-mediated ERp44 knock down. Immunoblot analysis reveals enhanced secretion of IL-23α^{wt} upon knock down of ERp44. Relative knock down of ERp44 and relative secretion of IL-23α^{wt} was determined form at least three independent

experiments (shown ±SEM). The ERp44 knock down was normalized to the ERp44 signal with the control siRNA (Ctrl siRNA, -), which was set to 100%. Statistical significance was calculated using a two-tailed unpaired t-test. ****$p<0.0001$, ***$p<0.001$ indicate statistically significant differences. **g** Immunoblot analysis of co-immunoprecipitated co-transfected hamster BiP or endogenous ERp44 with FLAG-tagged IL-23α^{wt}, IL-23α^{C14S}, IL-23α^{C22S} or IL-23$\alpha^{C14,22S}$. Relative binding was determined from at least six independent experiments (shown ±SEM) and normalized to the IL-23α^{wt} signal, which was set to 100% relative binding. Statistical significance was calculated using a two-tailed unpaired t-test. ns = not significant; *$p<0.05$, **$p<0.01$ indicate statistically significant differences.

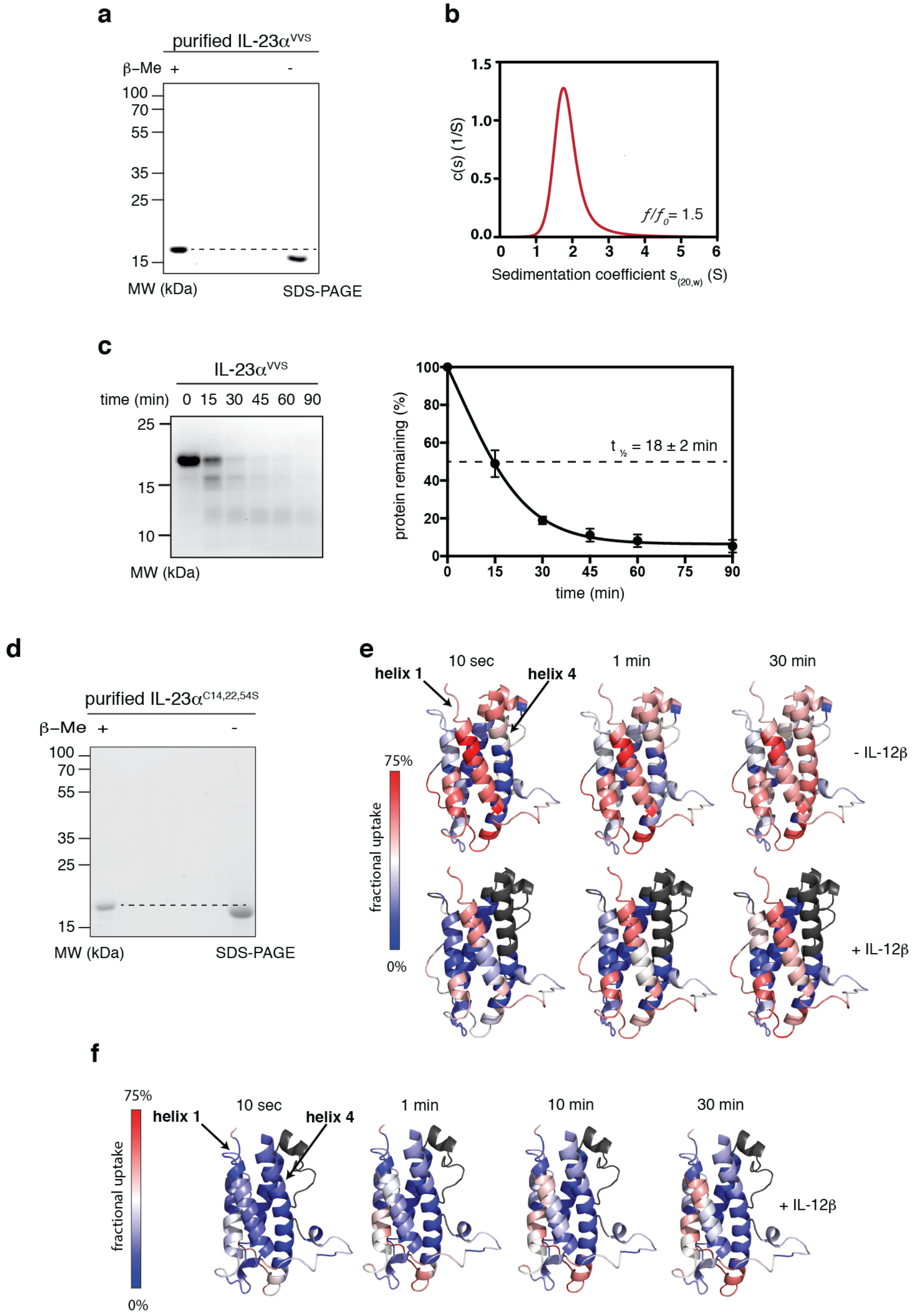

Supplementary Fig. 3 Folding status of recombinant IL-23α. **a** Analysis of recombinant IL-23α^{VVS} by (non-reducing) SDS-PAGE shows absence of covalent

misfolding and formation of an internal disulfide bond. **b** Analytical ultracentrifugation confirms a monomeric state of purified IL-23α^{VVS} ($MW_{monomer,\ calculated}$ = 18.9 KDa / MW_{auc} = 19.1 kDa) and a slightly elongated shape (f/f_0 = 1.5). **c** IL-23α^{VVS} is degraded with a half-life of 18 ± 2 min (SD) in partial proteolysis experiments with trypsin. The half-live from an exponential fit (black) of single data points (±SD, N=3) is shown. Source data are provided as a Source Data File. **d** Analysis of recombinant IL-23$\alpha^{C14,22,54S}$ (IL-23α^{SSS}) by (non-reducing) SDS-PAGE shows absence of covalent misfolding and formation of an internal disulfide bond. **e** Hydrogen/deuterium exchange (HDX) experiments of unpaired IL-23$\alpha^{C14,22,54S}$ *versus* IL-12β-paired IL-23^{C14,22S} (C54 is preserved in the heterodimer to allow quantitative complex formation). IL-23α is colored according to the differences in the measured HDX rates. Blue colors correspond to a lower (less flexible regions) and red colors to a higher (flexible regions) fractional uptake (grey: no sequence coverage in HDX measurements). **f** Hydrogen/deuterium exchange (HDX) experiments of IL-12β-paired IL-23α^{wt}. IL-23α is colored according to the differences in the measured HDX rates. Blue colors correspond to a lower (less flexible regions) and red colors to a higher (flexible regions) fractional uptake (grey: no sequence coverage in HDX measurements).

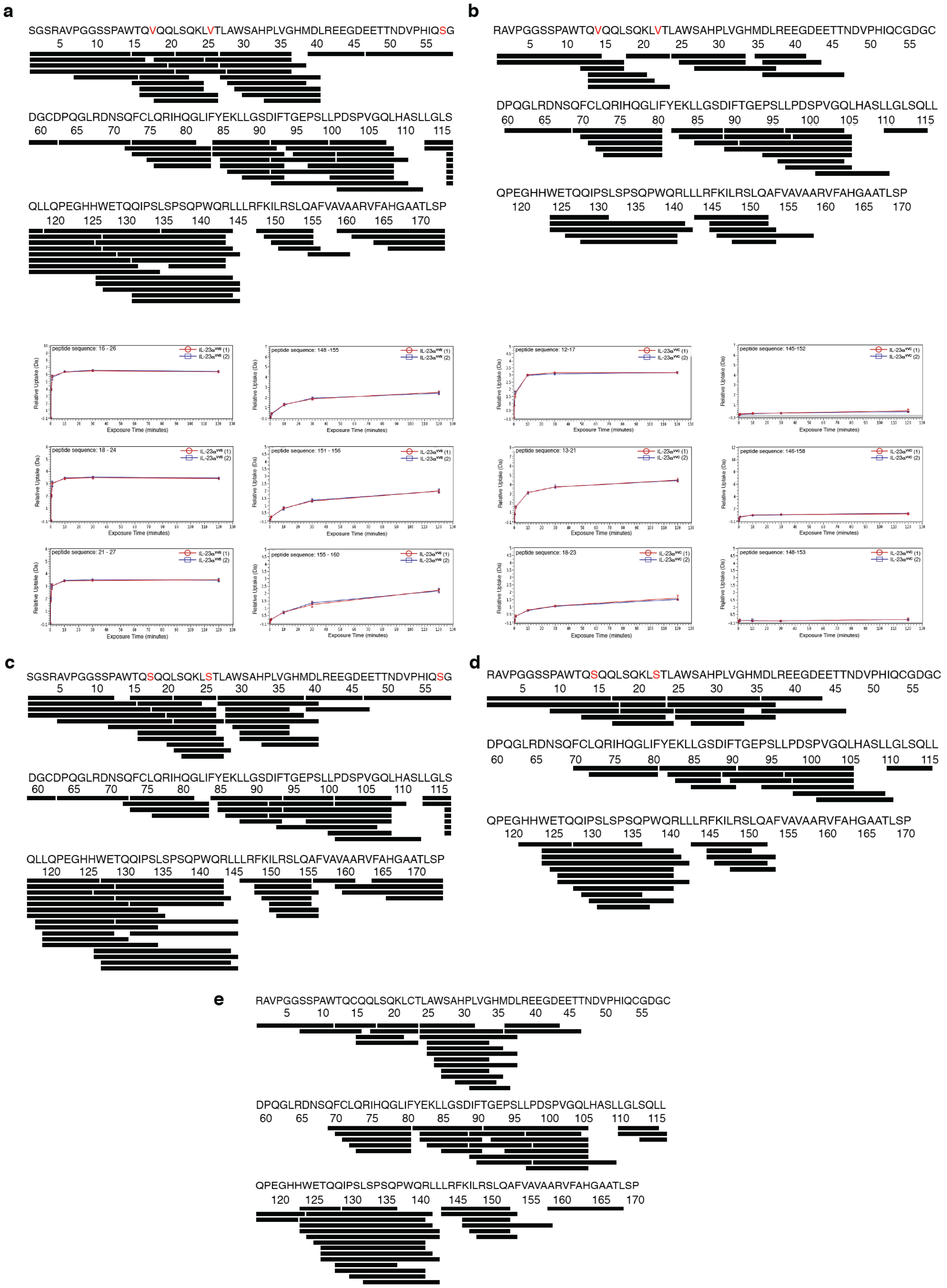

Supplementary Fig. 4 HDX data of IL-23α$^{VVS/VVC}$ and IL-23α$^{SSS/SSC}$. **a** Top: Peptide coverage map of IL-23α^{VVS}. Cysteine replacements in IL-23α^{VVS} are highlighted in red. Total coverage was 98.8% with 81 peptides and a redundancy of 5.35. Bottom:

Exchange graphs showing the relative deuterium uptake over the reaction time of three individual peptides from helix 1 (left) and 3 individual peptides from helix 4 (right). Each graph is labeled with the sequence of the respective peptide and its position within the primary sequence in the upper left corner. The error bars reflect the SD of three technical replicate measurements, red and blue display the two biological replicates. Helix 1 shows an increased exchange compared to helix 4 (red/blue: repeat 1/2). **b** Top: Peptide coverage map of IL-23α^{VVC}, purified as a heterodimer with IL-12β (cysteine 54 was present). Cysteine replacements in IL-23α^{VVC} are highlighted in red. Total coverage was 80% with 43 peptides and a redundancy of 3.20. Bottom: Exchange graphs showing the relative deuterium uptake over the reaction time of three individual peptides from helix 1 (left) and 3 individual peptides from helix 4 (right). Each graph is labeled with the sequence of the respective peptide and its position within the primary sequence in the upper left corner. The error bars reflect the SD of three technical replicate measurements, red and blue display the two biological replicates. **c** Peptide coverage map of IL-23α^{SSS}. Cysteine replacements in IL-23α^{SSS} are highlighted in red. Total coverage was 100% with 84 peptides and a redundancy of 5.64. **d** Peptide coverage map of IL-23α^{SSC}, purified as a heterodimer with IL-12β (cysteine 54 was present). Cysteine replacements in IL-23α^{SSC} are highlighted in red. Total coverage was 73.5% with 47 peptides and a redundancy of 3.90. **e** Peptide coverage map of IL-23α^{wt}, purified as a heterodimer with IL-12β. Total coverage was 85.9% with 67 peptides and a redundancy of 4.84.

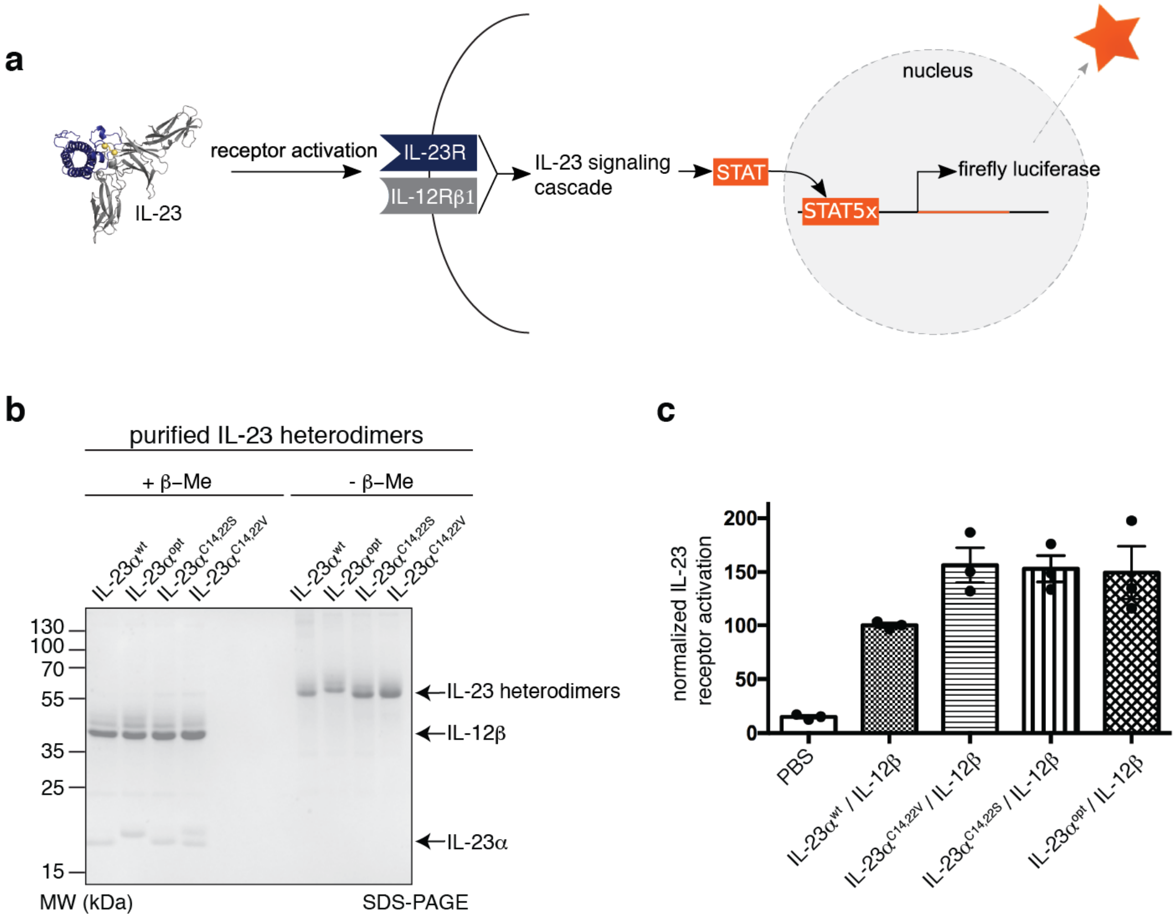

Supplementary Fig. 5 Biological functionality of IL-23 variants. **a** Schematic of the iLite® IL-23 receptor activation assay. The assay is based on a genetically engineered reporter gene cell line, which expresses the IL-23 receptor (composed of two receptor chains: IL-23R and IL-12Rβ1). IL-23 receptor activation is coupled to specific, proportional expression of Firefly Luciferase which serves as readout. A renilla luciferase reporter gene construct, under control of a constitutive promotor, is used for normalization. **b** Analysis of recombinant IL-23 complexes purified from mammalian cells by (non-reducing) SDS-PAGE shows formation of the disulfide-bridged complex. The double band pattern in IL-23$\alpha^{C14,22V}$ was due to incomplete His-tag cleavage. IL-12β was present in each complex. **c** Biological activity of recombinant IL-23 complexes. Induction of IL-23 signaling by the indicated IL-23 complexes (25 ng/ml final concentration) was assessed with the receptor activation assay described in **a**.

Relative receptor activation of each complex was calculated for at least three independent experiments (shown ±SEM) and normalized to the IL-23^{wt} complex signal, which was set to 100%.

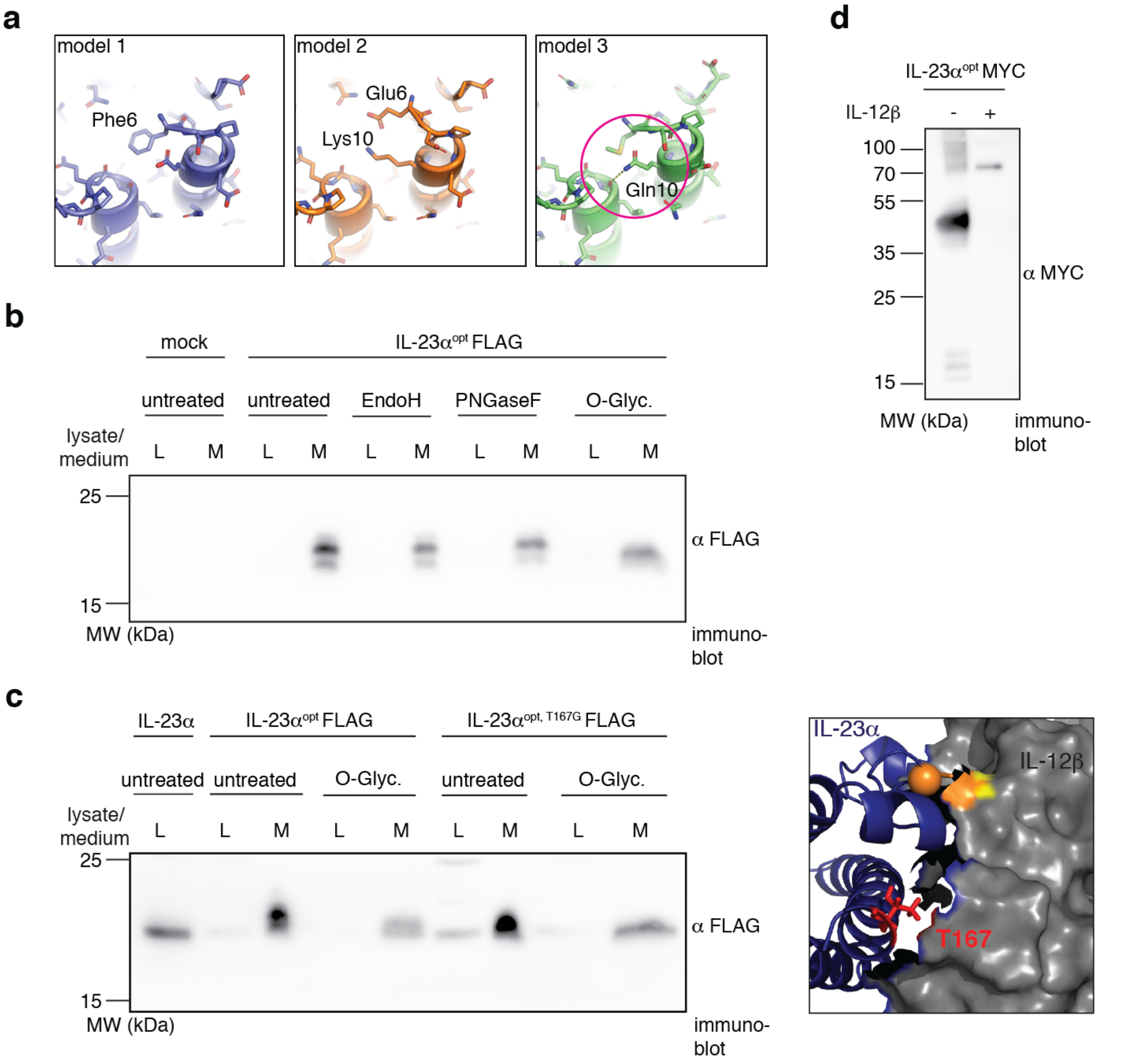

Supplementary Fig. 6 *In silico* design and *in vivo* and *in vitro* characterization of IL-23α^{opt}. **a** Design models of optimized IL-23α (IL-23α^{opt}). The three top models from the second design step where the N-terminus was extended by two residues are shown. We chose model 3 because it suggests a possibility that a hydrogen bond can form between residue Gln10 (magenta circle) and the neighboring helix. Model 1 has similar hydrophobic contacts as model 3, but Phe6 precluded the H-bond formation. Model 2 had a salt-bridge at the N-terminus, whereas hydrophobic packing would lead to more inter-helical stability. **b** IL-23α^{opt} becomes O-glycosylated upon independent secretion. Secreted FLAG-tagged IL-23α^{opt} from HEK 293T cells was treated with the indicated

de-glycosylation enzymes (O-Glyc.: mix of O-Glycosidase and α2-3,6,8 Neuraminidase as described in the method section) and analyzed on an immunoblot. **c** T167 was identified as an O-glycosylation site in IL-23α^{opt}. Replacing T167 by Gly (T167G) abolished O-glycosylation of FLAG-tagged IL-23α^{opt}. Secreted FLAG-tagged IL-23$\alpha^{opt, T167G}$ from HEK 293T cells was treated as in **b**. Residue T167 is located within the interface of IL-23α and IL-12β and becomes buried upon heterodimer formation, precluding O-glycosylation (model on the right; T167 is shown in red, the interchain disulfide bond-forming cysteine residues between IL-23α and IL-12β in orange/yellow). **d** Oxidation status of IL-23α^{opt} in the presence and absence of IL-12β. Cells were treated with Brefeldin A to retain proteins in cells.

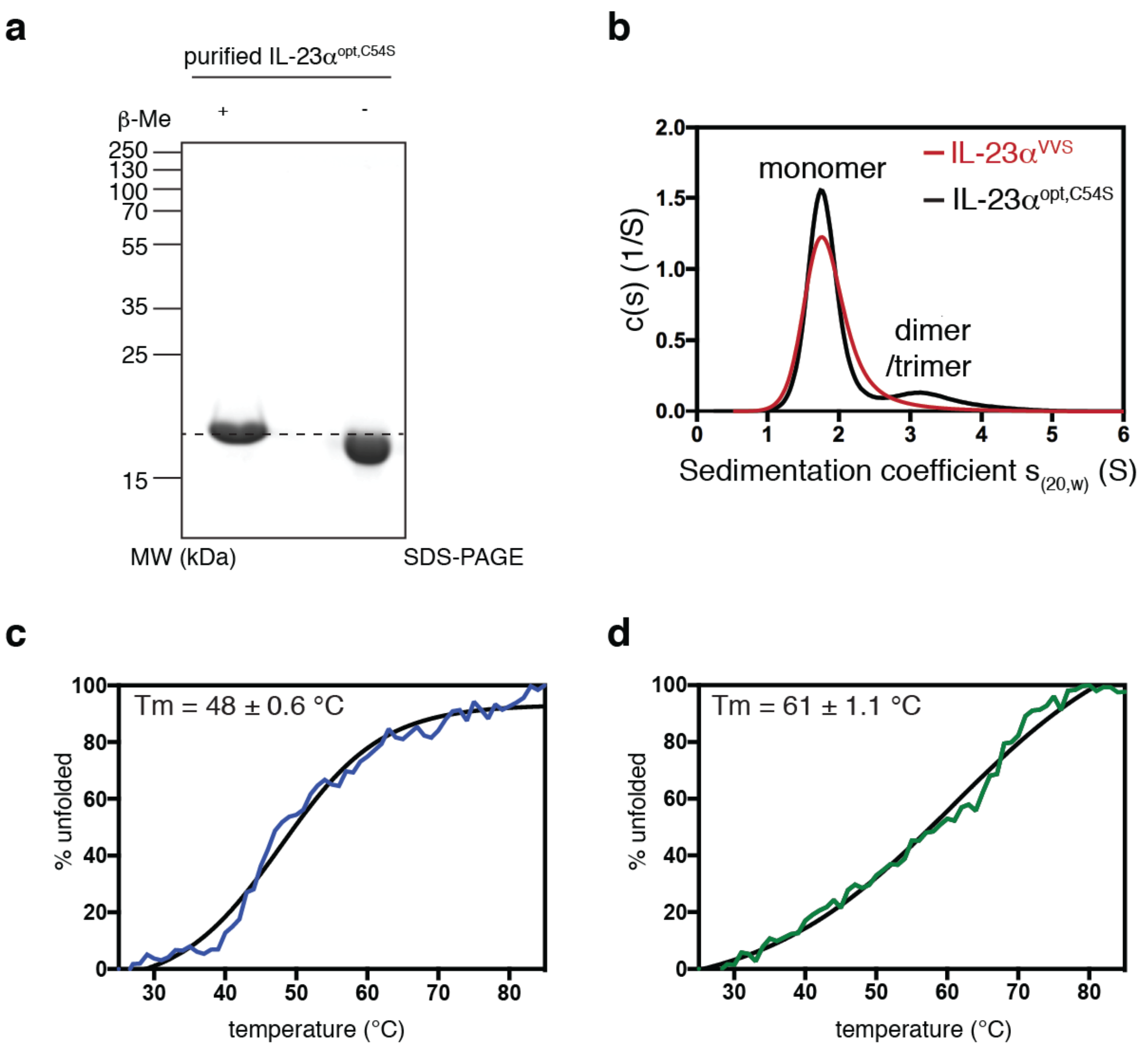

Supplementary Fig. 7 Folding status of IL-23α^{opt,C54S}. **a** Analysis of recombinant IL-23α^{opt,C54S} by (non-reducing) SDS-PAGE shows absence of covalent misfolding and formation of an internal disulfide bond. **b** Analytical ultracentrifugation confirms a predominantly monomeric state of recombinant IL-23α^{opt,C54S} ($MW_{monomer, calculated}$ = 18.9 KDa / MW_{auc} = 22.6 kDa) with low percentage of dimer or trimer formation (MW_{auc} = 56.7 kDa) and a slightly elongated shape (*f/f*$_0$ = 1.45). **c** Near-UV CD thermal transition measured at 260 nm of IL-23α^{VVS}**.** IL-23α^{VVS} unfolds cooperatively with a melting temperature (Tm) of 48 ± 0.6°C. **d** Near-UV CD thermal transition measured at 260 nm of IL-23α^{opt,C54S}. IL-23α^{opt,C54S} unfolds with a melting temperature of 61 ± 1.1°C and low cooperativity.

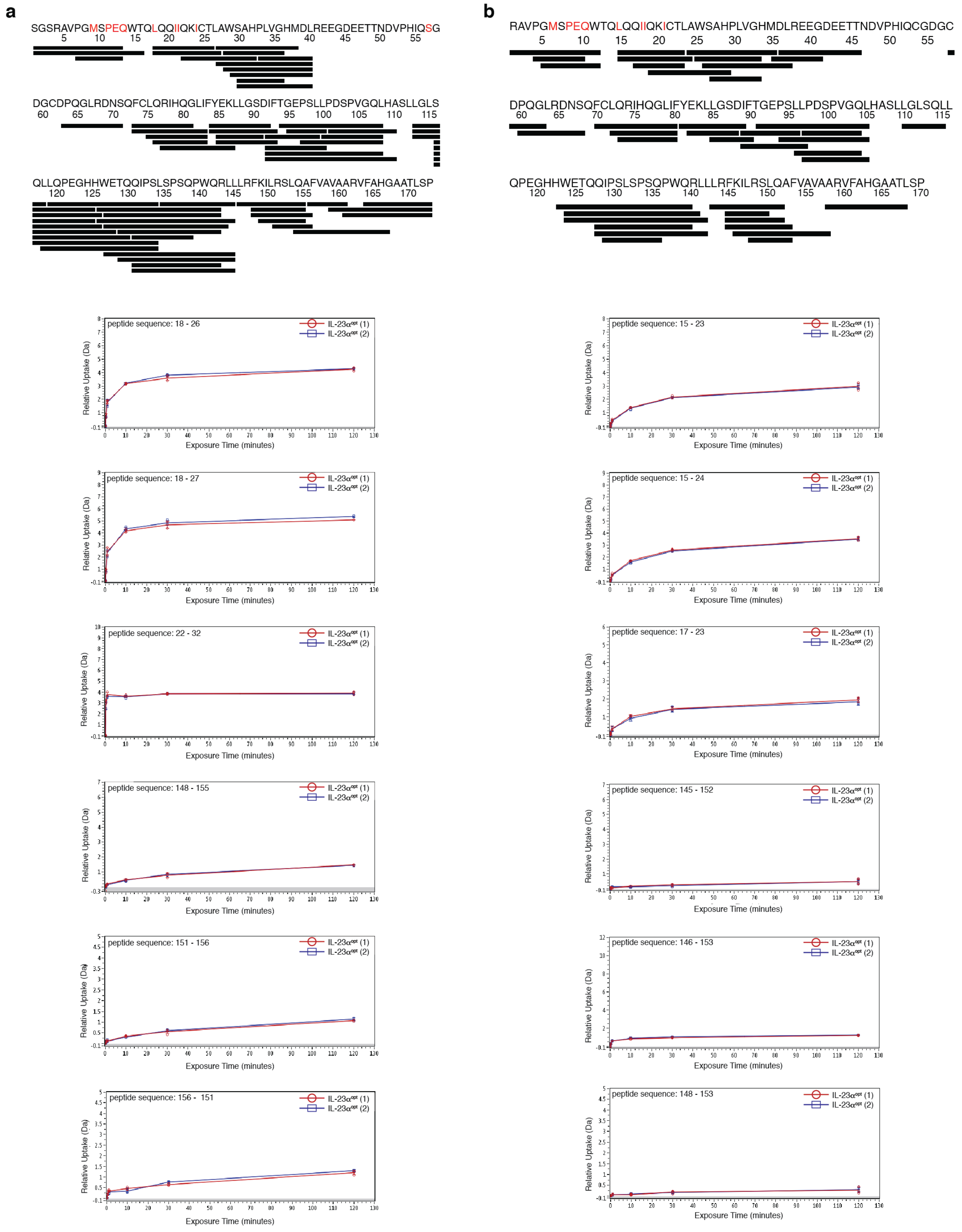

Supplementary Fig. 8 HDX data of IL-23α^{opt}. **a** Top: Peptide coverage map of IL-23α^{opt}. Mutations in comparison to IL-23α^{wt} and cysteine replacements of IL-23α^{opt,C54S} are highlighted in red. Total coverage was 85% with 64 peptides and a redundancy of 5.12. Bottom: Exchange graphs showing the relative deuterium uptake over the

reaction time of three individual peptides from helix 1 (top three panels) and 3 individual peptides from helix 4 (bottom three panels). Each graph is labeled with the sequence of the respective peptide and its position within the primary sequence in the upper left corner. The error bars reflect the SD of three technical replicate measurements, red and blue display the two biological replicates. **b** Top: Peptide coverage map of IL-23α^{opt}, purified as a heterodimer with IL-12β (cysteine 54 was present). Mutations in comparison to IL-23α^{wt} and cysteine replacements of IL-23α^{opt} are highlighted in red. Total coverage was 84.1% with 42 peptides and a redundancy of 2.92. Bottom: Exchange graphs showing the relative deuterium uptake over the reaction time of three individual peptides from helix 1 (top three panels) and 3 individual peptides from helix 4 (bottom three panels). Each graph is labeled with the sequence of the respective peptide and its position within the primary sequence in the upper left corner. The error bars reflect the SD of three technical replicate measurements, red and blue display the two biological replicates.

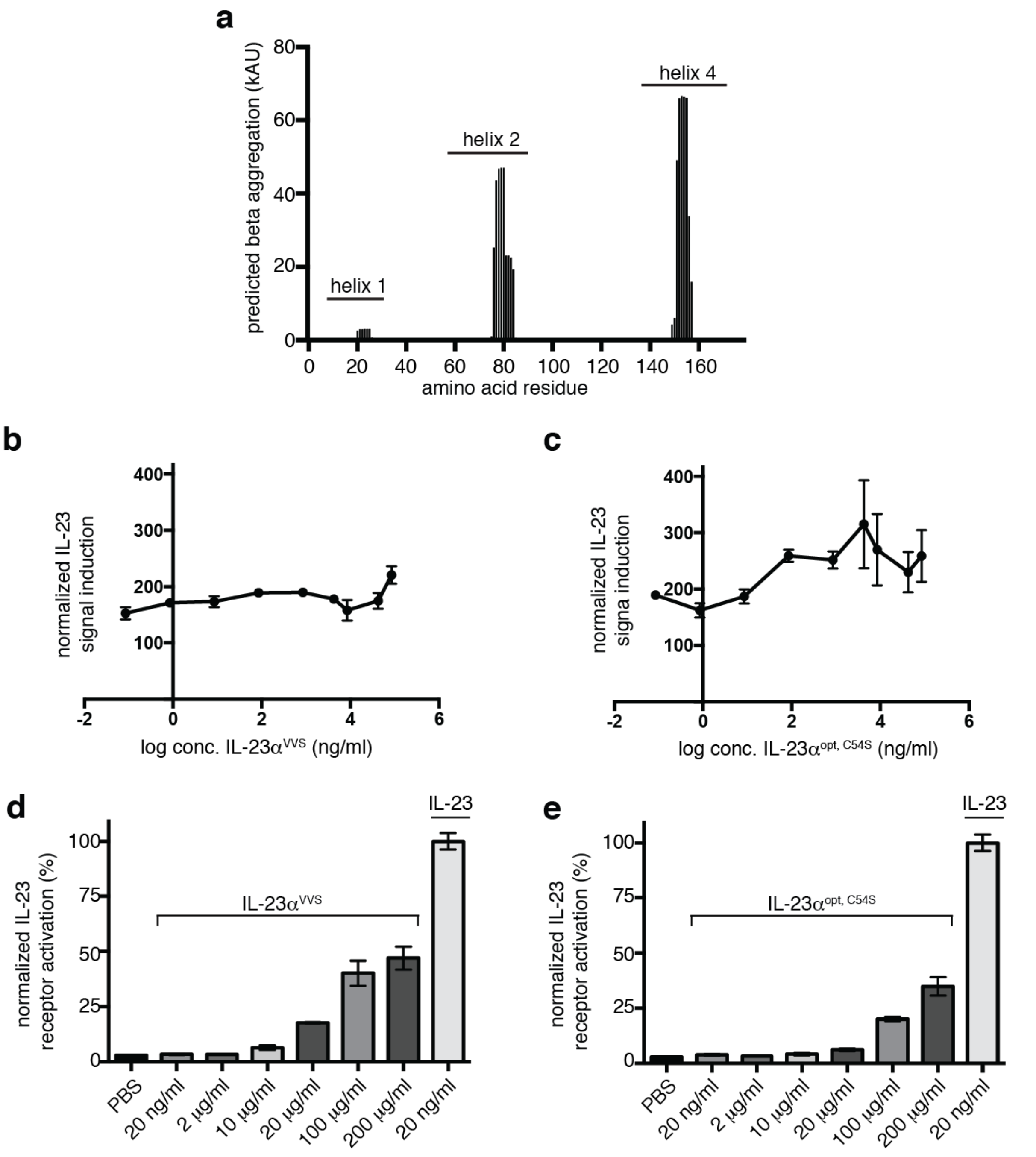

Supplementary Fig. 9 TANGO aggregation prediction and biological activity of IL-23α. **a** The TANGO algorithm[57] was used to calculate the β-aggregation potential for IL-23α^{wt}, which correlates with binding of pro-degradative BiP co-chaperones[35]. **b** Assessing neutralizing effects of recombinant IL-23α^{VVS} on IL-23 signaling using the iLite® assay. Molar ratios from 1:1 to 1:10.000 of IL-23:IL-23α^{VVS} were tested in three independent experiments (±SD). Source data are provided as a Source Data File. **c**

The same as in **b** but IL-23$\alpha^{opt,C54S}$ was used. Source data are provided as a Source Data File. **d** Assessing IL-23-like signaling by recombinant IL-23α^{VVS} using the iLite® assay. Indicated concentrations of unassembled recombinant IL-23α^{VVS} were compared to signal induction by (commercially available) IL-23. The IL-23α^{VVS} signal was normalized against signal induction by IL-23, which was set to 100% (±SD, N=3). Source data are provided as a Source Data File. **e** The same as in **d** but IL-23$\alpha^{opt,C54S}$ was used. Source data are provided as a Source Data File.

**IL-23α mutagenic primers (pSVL)**	
C14S (for)<br>C14S (rev)	CCTGGACTCAGAGTCAGCAGCTTTC<br>GAAAGCTGCTGACTCTGAGTCCAGG
C14V (for)<br>C14V (rev)	TGGACTCAGGTCCAGCAGCTTTCA<br>TGAAAGCTGCTGGACCTGAGTCCA
C22S (for)<br>C22S (rev)	AGAAGCTCAGCACACTGGCCTG<br>CAGGCCAGTGTGCTGAGCTTCT
C22V (for)<br>C22V (rev)	ACAGAAGCTCGTCACACTGGCCT<br>AGGCCAGTGTGACGAGCTTCTGT
C54S (for)<br>C54S (rev)	TGTTCCCCATATCCAGAGTGGAGATGGCTGTGA<br>TCACAGCCATCTCCACTCTGGATATGGGGAACA
C58S (for)<br>C58S (rev)	GTGTGGAGATGGCTCTGACCCCCAAG<br>CTTGGGGGTCAGAGCCATCTCCACAC
C70S (for)<br>C70S (rev)	CAACAGTCAGTTCTCCTTGCAAAGGATCC<br>GGATCCTTTGCAAGGAGAACTGACTGTTG
T167G (for)<br>T167G (rev)	GCCCATGGAGCAGCAGGCCTGAGTCCCGGCTCC<br>GGAGCCGGGACTCAGGCCTGCTGCTCCATGGGC
**IL-23α mutagenic primers (pcDNA 3.4 TOPO)**	
C14S (for)<br>C14S (rev)	GGACACAGAGCCAGCAGCT<br>AGCTGCTGGCTCTGTGTCC
C14V (for)<br>C14V (rev)	GGACACAGGTCCAGCAGCT<br>AGCTGCTGGACCTGTGTCC
C22S (for)<br>C22S (rev)	CAGAAGCTGAGTACACTGGCTTG<br>CAAGCCAGTGTACTCAGCTTCTG
C22V (for)<br>C22V (rev)	CAGAAGCTGGTCACACTGGCT<br>AGCCAGTGTGACCAGCTTCTG
**IL-23α mutagenic primers (pET21a)**	
W11Y (for)<br>W11Y (rev)	CAGCCCTGCCTACACTCAGAGTCA<br>TGACTCTGAGTGTAGGCAGGGCTG
W26Y (for)<br>W26Y (rev)	TCACAGAAGCTCTGCACACTGGCCTGGAGTGCACATCCACTAGTGGGACACA<br>TGTGTCCCACTAGTGGATGTGCACTCCAGGCCAGTGTGCAGAGCTTCTGTGA
**IL-12β mutagenic primers (pcDNA 3.4 TOPO)**	
STOP before His-tag(for)<br>STOP before His-tag(rev)	CCTTGTTCTGGCTCTTGAGAGAACCTGTACTTC<br>GAAGTACAGGTTCTCTCAAGAGCCAGAACAAGG
**Cloning primers**	
FLAG-tag insertion (pSVL vector) (for)<br>FLAG-tag insertion (pSVL vector) (rev)	TATACTCGAGGCCACCATGCTGGGG<br>TATAGAGCTCTTACTACTTGTCGTCGTCGTCCTTGTAGTC GGAGCCGGAGCCGGG
Myc-tag insertion (pSVL vector) (for)<br>Myc-tag insertion (pSVL vector) (rev)	TATACTCGAGGCCACCATGCTGGGG<br>TATACTCGAGTTACTACAGATCCTCTTCTGAGATGAGTTT TTGTTCGGAGCCGGAGCCGGGACTCAGGGTTGCTGCTCC
His-tag insertion IL-23α^{opt} (pET21a vector) (for)<br>His-tag insertion IL-23α^{opt} (pET21a vector) (rev)	TATAATATGGGTAGCCATCATCATCACCATCATGGTAGCGGTAGCGAAAATCT GTATTTTCAGAGCGGTAGCAGAGCTGTGCCTGGGATGAGCCCCGAGCAGTG G<br>TATACTCGAGTTACTAGGGACTCAGGGTTGC

Supplementary Table 1 Sequences of primers used in this study. Sequences are shown in a 5’ to 3’ orientation.

IL-23α^{wt}	ATGCTGGGATCTAGAGCTGTGATGCTGCTGCTCCTGCTGCCTTGGACAGCTCAGGGTAG<br>AGCTGTTCCCGGCGGATCTTCTCCAGCTTGGACACAGTGTCAGCAGCTGTCCCAGAAGCT<br>GTGCACACTGGCTTGGAGCGCTCATCCTCTCGTGGGCCACATGGACCTGAGAGAGGAAG<br>GCGACGAAGAGACTACCAACGACGTGCCCCATATCCAGTGTGGCGACGGCTGTGATCCT<br>CAGGGCCTGAGAGACAACTCCCAGTTCTGTCTGCAGCGGATCCACCAGGGACTGATCTT<br>CTACGAGAAGCTGCTGGGCTCCGACATCTTCACCGGCGAACCTTCTCTGCTGCCCGATTC<br>TCCTGTTGGCCAGCTGCATGCATCTCTGCTGGGACTGTCTCAGCTGCTGCAGCCTGAGG<br>GCCATCATTGGGAGACACAGCAGATCCCTTCTCTGTCCCCATCTCAGCCCTGGCAGAGAC<br>TGCTGCTGCGGTTCAAGATCCTGAGATCCCTGCAGGCCTTTGTGGCCGTGGCCGCTAGA<br>GTTTTTGCTCACGGTGCCGCTACACTGAGCCCCTGATGA
IL-23α^{opt}	ATGCTGGGATCTAGAGCTGTGATGCTGCTGCTCCTGCTGCCTTGGACAGCTCAGGGTAG<br>AGCAGTGCCTGGCATGTCTCCTGAGCAGTGGACACAGCTGCAGCAGATCATCCAGAAGA<br>TCTGCACCCTGGCTTGGAGCGCTCATCCTCTCGTGGGCCACATGGACCTGAGAGAGGAA<br>GGCGACGAAGAGACTACCAACGACGTGCCCCATATCCAGTGTGGCGACGGCTGTGATCC<br>TCAGGGCCTGAGAGACAACTCCCAGTTCTGTCTGCAGCGGATCCACCAGGGACTGATCTT<br>CTACGAGAAGCTGCTGGGCTCCGACATCTTCACCGGCGAACCTTCTCTGCTGCCCGATTC<br>TCCTGTGGGCCAGCTGCATGCTTCTCTGTTGGGACTGTCTCAGCTGCTCCAGCCTGAGG<br>GCCATCATTGGGAGACACAGCAGATCCCTTCTCTGTCCCCATCTCAGCCCTGGCAGAGAC<br>TGCTGCTGCGGTTCAAGATCCTGAGATCCCTGCAGGCCTTTGTGGCCGTGGCCGCTAGA<br>GTTTTTGCTCACGGTGCCGCTACTCTGTCTCCTGGCGGCGAGAACCTGTACTTCCAGAGC<br>CACCACCACCATCACCACTGATGA
IL-23α^{C14,22S}	ATGCTGGGATCTAGAGCTGTGATGCTGCTGCTCCTGCTGCCTTGGACAGCTCAGGGTAG<br>AGCTGTTCCCGGCGGATCTTCTCCAGCTTGGACACAGAGCCAGCAGCTGTCCCAGAAGC<br>TGAGTACACTGGCTTGGAGCGCTCATCCTCTCGTGGGCCACATGGACCTGAGAGAGGAA<br>GGCGACGAAGAGACTACCAACGACGTGCCCCATATCCAGTGTGGCGACGGCTGTGATCC<br>TCAGGGCCTGAGAGACAACTCCCAGTTCTGTCTGCAGCGGATCCACCAGGGACTGATCTT<br>CTACGAGAAGCTGCTGGGCTCCGACATCTTCACCGGCGAACCTTCTCTGCTGCCCGATTC<br>TCCTGTTGGCCAGCTGCATGCATCTCTGCTGGGACTGTCTCAGCTGCTGCAGCCTGAGG<br>GCCATCATTGGGAGACACAGCAGATCCCTTCTCTGTCCCCATCTCAGCCCTGGCAGAGAC<br>TGCTGCTGCGGTTCAAGATCCTGAGATCCCTGCAGGCCTTTGTGGCCGTGGCCGCTAGA<br>GTTTTTGCTCACGGTGCCGCTACACTGAGCCCCTGATAA
IL-23α^{C14,22V}	ATGCTGGGATCTAGAGCTGTGATGCTGCTGCTCCTGCTGCCTTGGACAGCTCAGGGTAG<br>AGCTGTTCCCGGCGGATCTTCTCCAGCTTGGACACAGGTCCAGCAGCTGTCCCAGAAGC<br>TGGTCACACTGGCTTGGAGCGCTCATCCTCTCGTGGGCCACATGGACCTGAGAGAGGAA<br>GGCGACGAAGAGACTACCAACGACGTGCCCCATATCCAGTGTGGCGACGGCTGTGATCC<br>TCAGGGCCTGAGAGACAACTCCCAGTTCTGTCTGCAGCGGATCCACCAGGGACTGATCTT<br>CTACGAGAAGCTGCTGGGCTCCGACATCTTCACCGGCGAACCTTCTCTGCTGCCCGATTC<br>TCCTGTTGGCCAGCTGCATGCATCTCTGCTGGGACTGTCTCAGCTGCTGCAGCCTGAGG<br>GCCATCATTGGGAGACACAGCAGATCCCTTCTCTGTCCCCATCTCAGCCCTGGCAGAGAC<br>TGCTGCTGCGGTTCAAGATCCTGAGATCCCTGCAGGCCTTTGTGGCCGTGGCCGCTAGA<br>GTTTTTGCTCACGGTGCCGCTACACTGAGCCCCTGATAA
IL-12β	ATGTGTCATCAGCAGCTGGTCATCAGCTGGTTCTCCCTGGTGTTCCTGGCCTCTCCTCTG<br>GTGGCTATCTGGGAGCTGAAGAAAGACGTGTACGTGGTGGAACTGGACTGGTATCCTGA<br>CGCTCCTGGCGAGATGGTGGTGCTGACCTGTGATACCCCTGAAGAGGACGGCATCACCT<br>GGACACTGGACCAGTCCTCTGAGGTGCTCGGCTCTGGCAAGACCCTGACCATCCAAGTG<br>AAAGAGTTTGGCGACGCCGGCCAGTACACCTGTCACAAAGGCGGAGAGGTGCTGTCCCA<br>CAGTCTGCTGCTGCTCCACAAGAAAGAGGATGGAATCTGGTCCACCGACATCCTGAAGGA<br>CCAGAAAGAGCCCAAGAACAAGACCTTCCTGAGATGCGAGGCCAAGAACTACTCCGGCC<br>GGTTCACATGTTGGTGGCTGACCACCATCAGCACCGACCTGACCTTCTCCGTGAAGTCCT<br>CCAGAGGCTCTAGCGATCCTCAGGGCGTTACATGTGGCGCTGCTACCCTGTCTGCTGAG<br>AGAGTGCGGGGCGACAACAAAGAATACGAGTACTCCGTGGAATGCCAAGAGGACTCCGC<br>CTGTCCTGCCGCTGAAGAGTCTCTGCCCATCGAAGTGATGGTGGACGCCGTGCACAAGC<br>TGAAGTACGAGAACTACACCTCCAGCTTTTTCATCCGGGACATCATCAAGCCCGATCCTC<br>CAAAGAACCTGCAGCTGAAGCCTCTGAAGAACTCCAGACAGGTGGAAGTGTCCTGGGAG<br>TACCCCGACACCTGGTCTACCCCTCACTCCTACTTCAGCCTGACCTTTTGCGTGCAAGTG<br>CAGGGCAAGTCCAAGCGCGAGAAAAAGGACCGGGTGTTCACCGACAAGACCTCCGCCAC<br>CGTGATCTGCAGAAAGAACGCCTCCATCTCCGTCAGAGCCCAGGACCGGTACTACTCCA<br>GTTCTTGGAGCGAGTGGGCCTCCGTGCCTTGTTCTGGCTCTTGA

Supplementary Table 2 Sequences of *Cricetulus griseus* codon-optimized constructs used in this study. Sequences are shown in a 5’ to 3’ orientation.
